# Chemically Recyclable and Tunable Polyolefin‐Like Multiblock Copolymer Adhesives

**DOI:** 10.1002/anie.202513286

**Published:** 2025-09-08

**Authors:** Yucheng Zhao, Ethan C. Quinn, Megan E. Battson, Emma M. Rettner, Joel Miscall, Nicholas A. Rorrer, Eugene Y.‐X. Chen, Garret M. Miyake

**Affiliations:** ^1^ Department of Chemistry Colorado State University Fort Collins CO 80523 USA; ^2^ School of Materials Science and Engineering Colorado State University Fort Collins CO 80523 USA; ^3^ Renewable Resources and Enabling Sciences Center National Renewable Energy Laboratory Golden CO 80401 USA; ^4^ BOTTLE Consortium Golden CO 80401 USA

**Keywords:** Adhesives, Chemically recyclable, Multiblock, Multilayer materials, Polyethylene‐like

## Abstract

Adhesives are important in creating multilayer products, such as in packaging and construction. Most current hot‐melt adhesives such as poly(ethylene‐co‐vinyl acetate) (EVA) and polyurethanes lack chemical recyclability and do not easily de‐bond, complicating recycling. Here, we achieved tunable adhesive properties of chemically recyclable polyolefin‐like multiblock copolymers through regulating the incorporation of crystalline hard blocks, amorphous soft blocks, and ester content highlighted by adhesive strengths up to 6.80 MPa. We further demonstrated applications of these adhesives in multi‐layer films and showed that the adhesives can be readily de‐bonded, recovered, and chemically recycled. Overall, this study into structure–property relationships, including effects of crystallinity, soft‐block incorporation, and ester content of the multiblock copolymers on adhesive properties, has resulted in a modular hot‐melt adhesive platform that exhibits chemical recyclability for virgin‐quality adhesive re‐generation, reprocessability for repeated reuse of the recovered adhesive, and tunability for designing adhesives with a wide range of low to high (up to ∼80% stronger than EVA) adhesion strengths.

## Graphical abstract

We report chemically recyclable PE‐like multiblock copolymer adhesives with tunable cohesion and adhesion. By controlling crystallinity, ester content, and block structure, these materials achieve up to ∼80% stronger adhesion than EVA, while enabling depolymerization, reuse, and multilayer deconstruction—offering a sustainable and high‐performance HMA platform.

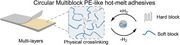

Adhesives have been recognized as an indispensable component in a wide range of industrial applications.^[^ ^1^ ^]^ In particular, their ability to form strong and continuous bonds offers distinct advantages over mechanical fasteners such as nails and screws in sectors including packaging, transportation, construction, lumber, and textiles.^[^ ^2^, ^3^, ^4^ ^]^ Unfortunately, at the end of their service life, adhesives often end up in landfills.^[^ ^5^ ^]^ This polluting practice is because it can be difficult to remove and recover adhesives from the products they hold together. In addition, adhesive residue can contaminate recycling streams and lead to the loss of valuable, multi‐component materials. For example, widely used poly(ethylene‐*co*‐vinyl acetate) (EVA) adhesives lack chemical recyclability, posing environmental concerns.^[^ ^6^ ^]^ These unsustainable challenges show the need for new adhesives that can be recovered and chemically recycled, contributing to addressing the global plastic waste problem.

To tackle these issues, researchers have explored sustainable adhesives using innovative strategies,^[^ ^4^, ^7^, ^8^, ^9^, ^10^ ^]^ including dynamic crosslinking,^[^ ^11^ ^]^ supramolecular thermosets,^[^ ^12^, ^13^, ^14^ ^]^ biobased adhesives,^[^ ^13^, ^14^ ^]^ or molecularly engineered adhesives.^[^ ^15^, ^16^, ^17^, ^18^, ^19^, ^20^ ^]^ Among plastics, polyolefins are the most widely used, making them particularly attractive targets for adhesive development. For instance, post‐functionalizing waste polyethylene (PE) by introducing oxygen functionality (e.g., ester),^[^ ^21^, ^22^, ^23^, ^24^, ^25^, ^26^, ^27^ ^]^ can yield functionalized PE‐like polyolefins with enhanced noncovalent interactions to bolster the interfacial bonding strength of adhesives. PE‐like polyesters have gained increasing attention as these ester linkages give the materials closed‐loop recyclability,^[^ ^28^, ^29^ ^]^ allowing them to be broken down chemically and reformed after use.^[^ ^30^, ^31^ ^]^ However, high ester content (>2%;, e.g., EVA contains 12% ester groups) can reduce the thermal and chemical stability of the adhesive (e.g., deacetylation of EVA occurs at 150 °C).^[^ ^32^ ^]^ Additionally, a persistent scientific challenge is the asynchronous control of cohesive strength and interfacial bonding strength, complicating efforts to simultaneously optimize these key properties.^[^ ^33^ ^]^ We hypothesize that addressing these issues could be achieved by manipulation of the internal structure and phase behavior of adhesives. Specifically, we believe that through strategically designed physical crosslinking networks, which enhance cohesive bonding within the PE‐like adhesives, combined with tuning of melt viscosity to enhance interfacial bonding to substrate, will synergistically enhance adhesive performance and recyclability.

Our interest in chemically recyclable plastics has leveraged the step‐growth polymerization of coupling diols to produce ester‐linked polyolefin‐like multiblock polymers, which can undergo hydrogenative depolymerization and dehydrogenative repolymerization using the same ruthenium pincer catalyst.^[^ ^34^ ^]^ With significantly reduced ester content compared to recent polyester adhesives, we hypothesized these multiblock copolymers would exhibit enhanced thermal stability while maintaining desirable adhesive properties and the capability to chemically debond and depolymerize into their original building blocks after use. These materials also possess a unique multiblock structure from both (crystalline) hard and (amorphous) soft PE segments. Such materials share structural similarities with olefin block copolymers (OBCs), which have demonstrated promising performance in adhesive applications.^[^ ^35^, ^36^, ^37^ ^]^ Recent studies have highlighted the ability to finely tune adhesive strength through the strategic incorporation of segments with differing chemical characteristics.^[^ ^38^ ^]^ Inspired by recent advances with biodegradable poly(3‐hydroxybutyrate) adhesive systems,^[^ ^8^ ^]^ we envisioned that carefully controlling the physical crosslinking between hard and soft segments in PE‐like multiblock copolymers could enhance cohesion strength and significantly improve melt viscosity tunability (Figure 1). Furthermore, these adhesives would be prone to chemical recyclability, a particularly valuable feature for recycling adhesives, enabling facile de‐bonding from attached substrates, complex assemblies, or mixed plastic waste streams. Guided by these hypotheses, this work demonstrates that multiblock copolymer hot‐melt adhesives can simultaneously achieve excellent adhesive performance and recyclability. This desired property was further highlighted by the creation of multilayered materials that could be deconstructed, and the component materials recovered.

**Figure 1:**
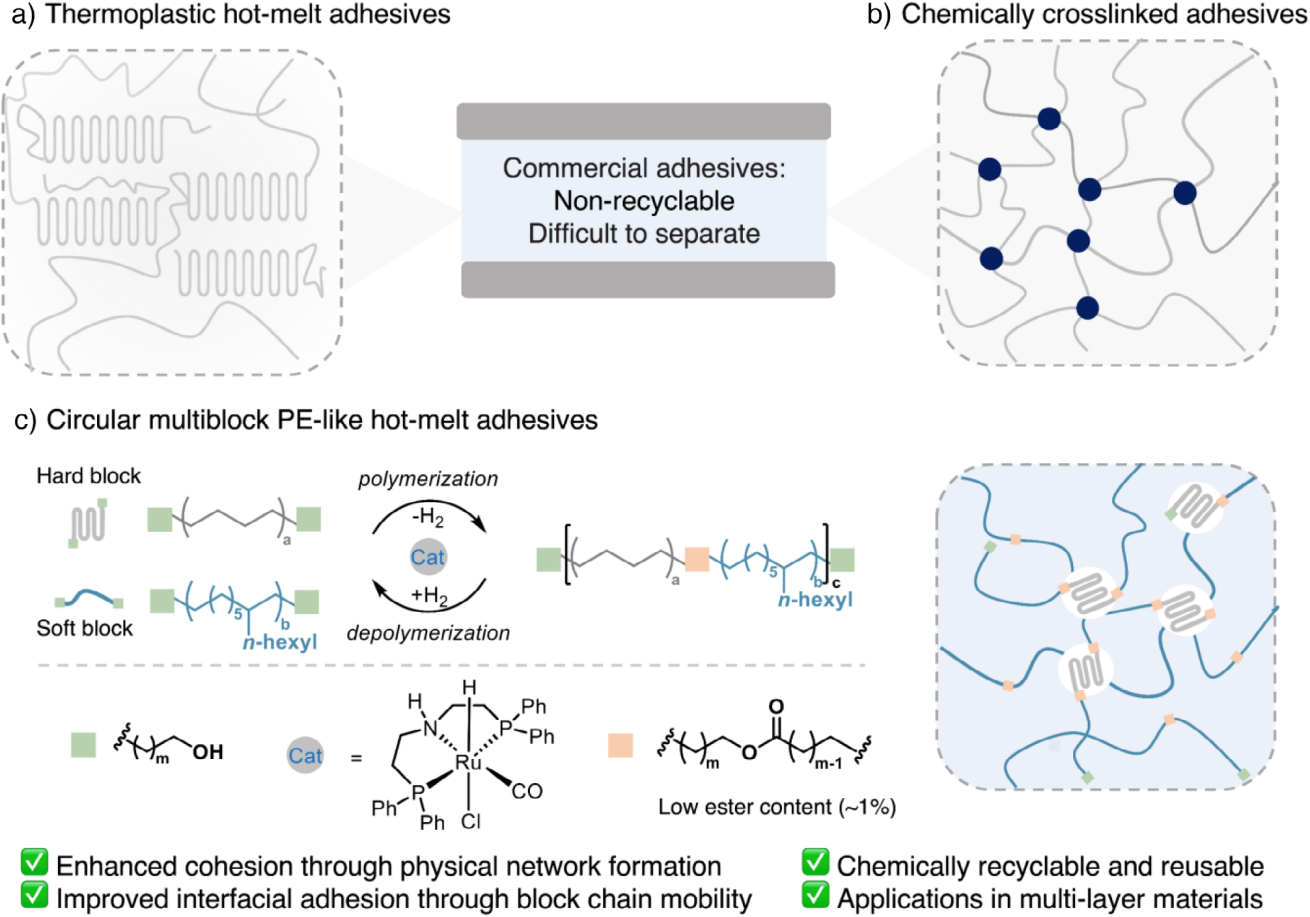
Representative commercial adhesives and their adhesion mechanisms. a) Thermoplastic hot‐melt adhesives relying on noncovalent interactions; b) Chemically crosslinked commercial adhesives with irreversible covalent networks; c) Chemically recyclable multiblock PE‐like adhesives, featuring enhanced cohesion through physical crosslinking networks and improved interfacial adhesion through promoted interfacial contact.

To test our hypotheses, we polymerized alcohol functionalized oligomeric building blocks to synthesize multiblock polymers with low ester linkage content and tested their performance as adhesives. Hard blocks (HB) created crystalline regions that provided mechanical strength and physical network, while soft blocks (SB) with flexible side chains modulated crystallinity and chain mobility. As shown in Figure 2, the hard–soft (HS) multiblock polymers (HS0–HS100, with the number denoting the %HB content) were synthesized via ruthenium‐catalyzed (Ru‐MACHO) acceptorless dehydrogenative polymerization by varying the feed ratio of HB (0%, 20%, 40%, 60%, 80%, and 100%).^[^ ^34^ ^]^ These polymers possessed weight‐average molecular weight (*M*_w_) ranging from 57.5 to 90.3 kDa and dispersities (*Ð*) of 2.31–3.90 (Table S1, Figures S1–S11). These PE‐like polymers contain much lower ester content (7.0–11.5 per 1000 carbon; ∼1%, Figure 2a) compared to EVA (126 per 1000 carbon; ∼12%, Figure 2a). Notably, these polymers exhibit a high melting transition temperature (*T*_m_ = 108–123 °C) and decomposition temperatures (*T*_d,5%_ >383 °C), demonstrating better thermal stability than commercial EVA (Figure 2b).^[^ ^32^ ^]^ This enhanced thermal stability suggests a broader operational temperature range.

**Figure 2:**
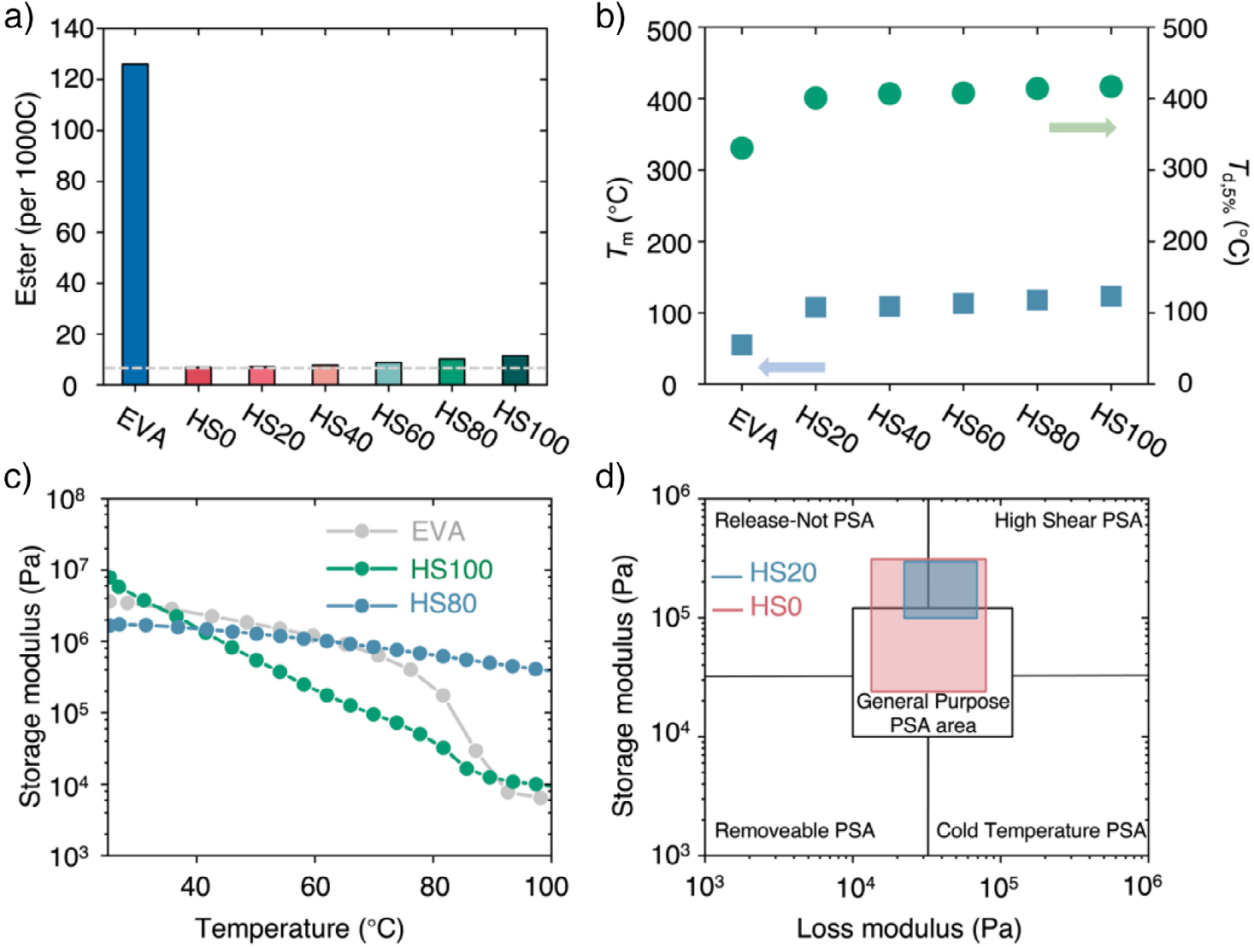
Physical, thermal, and mechanical properties of EVA and multiblock polymers HS100‐HS0. a) The ester content (per 1000 carbon) calculated based on ^1^H NMR analysis. Dashed line indicates the ester content as 10 per 1000 carbon. b) Melting (*T*_m_) and decomposition temperatures (*T*_d,5%_). c) Storage modulus of EVA, HS100, and HS80 from 25–100 °C. d) Chang's viscoelastic window of general pressure sensitive adhesive (PSA), HS0, and HS20.

To uncover the structure–property relationships between soft block content and adhesion strength, we first measured the HS multiblock copolymer's physical, thermal, and mechanical properties. As viscoelastic properties strongly influence adhesive behavior,^[^ ^8^ ^]^ we performed temperature sweeps on HS100‐HS0 from 25–100 °C (Figure 2c). Crystalline phases in semi‐crystalline PE act as physical crosslinking sites, which facilitate energy dissipation and enhance toughness.^[^ ^39^ ^]^ The storage modulus (*G*″) of EVA and HS100 decreased with increasing temperature, consistent with entangled, high‐molecular‐weight polymer having few physical crosslinks at elevated temperatures. With the incorporation of soft blocks, the *G*″ of HS80, HS60, and HS40 remained within a narrower range compared to HS100, indicating network formation driven by microphase separation of their blocky architectures. These materials thus have potential applications in hot‐melt adhesives, with tunable cohesion and interfacial adhesion properties. Upon incorporating higher SB content, HS20 and HS0 exhibited distinct viscoelastic characteristics: low storage modulus at high frequencies and high storage modulus at low frequencies (Figure 2d). These viscoelastic properties are ideal for pressure‐sensitive adhesives (PSA) used in applications like tapes and sticky notes. Chang previously proposed a viscoelastic window concept to categorize different types of PSAs based on their *G* ^″^ and loss moduli (*G″*) at high (100 rad s^−1^) and low frequencies (0.01 rad s^−1^).^[^ ^40^ ^]^ According to this classification, the central region represents general‐purpose PSAs, HS0, and HS20 fell within the high‐shear PSA region, highlighting their suitability for specialized adhesive applications. With these properties in hand, we then looked to measure the adhesion strength of these chemically recyclable PE‐like copolymers. HS100, an ester‐linked PE‐like polymer without SB incorporation, exhibited significantly higher adhesion strength to aluminum (4.70 MPa) than commercial polyethylene samples with various topologies (Figure 3a). This result suggests that incorporating a small amount of ester linkages not only enhances the recyclability of PE but also increases adhesive properties. Notably, HS100's adhesion was comparable to that of EVA (3.80 MPa; Figure 3a, Table S2), suggesting that this chemically recyclable PE‐like copolymer could be a replacement for EVA.

**Figure 3:**
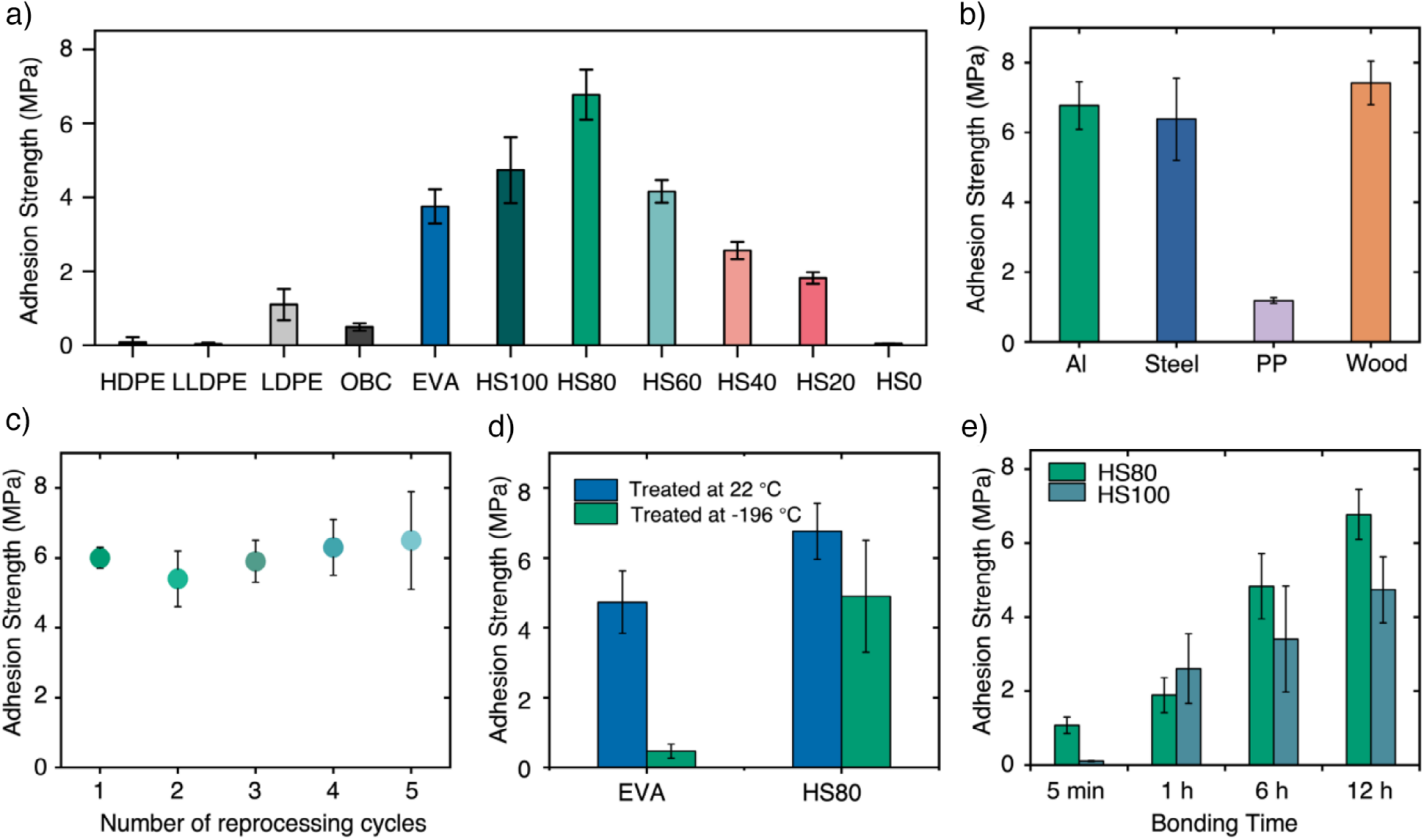
Chemically recyclable multiblock copolymers as hot‐melt adhesives. a) Adhesion strength (on aluminum) of commercial PEs, olefin block copolymer (OBC), EVA, and PE‐like polymers HS100–HS0. b) Adhesion strength of HS80 on various substrates. c). Adhesion strength of HS80 after reprocessing for 1–5 times. d) Adhesion strength of EVA and HS80 after treatment at room temperature or liquid nitrogen for 1 h. e) Adhesion strength as a function of bonding time for HS80 and HS100.

To further investigate how physical crosslinking introduced by SB incorporation influences adhesive performance, we characterized the SB content, ester content, and thermomechanical properties of HS80‐HS0 to contextualize their adhesion behavior. When 20 mol% soft content was introduced, HS80 showed higher adhesion strength (6.80 MPa) than HS100 and EVA. The adhesion strength further was modulated from 4.20 MPa (HS60), to 2.60 MPa (HS40), to 1.80 MPa (HS20), and finally to 0.04 MPa (HS0) as SB content increased. These tunable adhesive properties demonstrate that these polymers can range from permanent adhesives to PSAs based on SB composition.

HS80, the strongest adhesive among these polymers, exhibits strong adhesion to aluminum, steel, and wood (> 6.0 MPa), and moderate adhesion to polypropylene (PP) and high‐density polyethylene due to their low surface energy and slick surfaces (Figures 3b and S12, Table S3). Reusability tests of HS80 through reprocessing under defined conditions (22 °C; mechanically removing adhesive, melting, and adhering substrates again) revealed a consistent shear strength of ∼6 MPa across five reuse cycles (Figure 3c). We also evaluated the adhesion performance of HS80 after treatment at low temperature (−196 °C, liquid nitrogen) for 1 h, immediately followed by testing at room temperature. HS80 exhibited only a modest decrease (<25%) in adhesion strength, which is significantly superior compared to EVA's dramatic decrease (∼90%, Figure 3d), indicating HS80's superior low‐temperature performance when compared to EVA. Viscosity measurements of HS80 further showed no significant decline after three heat–cool cycles (Figure S13), confirming the robust reusability and thermal stability of this adhesive. Hence, this molecular design of incorporating soft blocks into low‐ester content chemically recyclable polymers efficiently transmits mechanical forces across adherends, achieving superior adhesion strength. This design also allows tunable shear strength by simply varying HB‐to‐SB from 0 to 100.

In the real‐world use of adhesives some bonds need to be created quickly (short set time) while some need to be able to be manipulated before creating a strong bond (long open time). To investigate the open and set times of these chemically recyclable hot‐melt adhesives, a series of time studies were conducted (Figure 3e). Both HS80 and HS100 exhibited increased adhesion over longer bonding times due to enhanced interfacial contact and potential mechanical interlocking. However, HS80 demonstrated significantly better performance at just 5 min (1.6 MPa) versus HS100 (0.05 MPa), and maintained a nearly constant degree of crystallinity (*X*_c_) across all time points (38%–40%). In contrast, HS100 showed a decrease in *X*_c_ from 67% to 50% (Figures S14 and S15). These results further suggest that incorporating soft blocks accelerates interfacial contact, promotes crystal reformation, and enables a diverse range of set and open times. To investigate the bonding properties of these adhesives, we determined the zero‐shear viscosity and plotted it against adhesion strength (Figure S16). HS80 showed a lower melt viscosity than HS100 and higher adhesion strength, pointing to enhanced chain mobility and improved surface contact. Furthermore, *X*_c_ was tuned from 65% (HS100) to 46% (HS80), increasing the amorphous domains that promote contact with surfaces—an effect consistent with the observed correlation between adhesion strength and crystallization behavior.^[^ ^34^, ^41^ ^]^ When increasing SB content (HS60, HS40, and HS20), the *X*_c_ dropped below 40%, leading to lower cohesion within the polymer network and therefore lower adhesion strength (Figures 3a and S17). An independent relationship between molecular weight (*M*_w_) and adhesion strength was observed for both HS80 and HS100 across a range of 41.7–96.6 kDa (Figure S18). In contrast, a positive correlation was found between toughness and adhesion strength, likely due to more efficient mechanical force transmission across the cohesive polymer network (Figure S19). These results further indicate that the incorporation of SB could tune melt viscosity to facilitate rapid interfacial contact, while allowing for quick formation of cohesive structures by constructing a network.

The influence of ester content was investigated to further uncover the structure–property relationships in these HS copolymers. We synthesized HS80 adhesives using building blocks with different molecular weights (HB and SB at 6 and 10 kDa, respectively) to modulate the ester incorporation. Their *T*_m_ (118–130 °C) and Young's modulus (280–380 MPa) were increased with the increasing of block chain length from 2, 6, and 10 kDa (Figures 4a and S24–S26). Meanwhile, the crystal size decreased (Figures S27 and S28) while the overall *X*_c_ remained similar, indicating the critical role of ester content and block length in the property regulation. Furthermore, a clear trend for adhesive property emerged: adhesives containing 2.0 to 2.6 to 10.2 ester per 1000 carbon exhibited adhesive strengths from 1.30 to 6.80 MPa, confirming that ester content critically affects adhesion in PE‐like materials (Figure 4b). Although the ester content was lower than that of functionalized PE samples (126 per 1000 carbon for EVA), the multiblock topology yielded comparable adhesion strength while maintaining more desirable PE‐like thermal properties.

**Figure 4:**
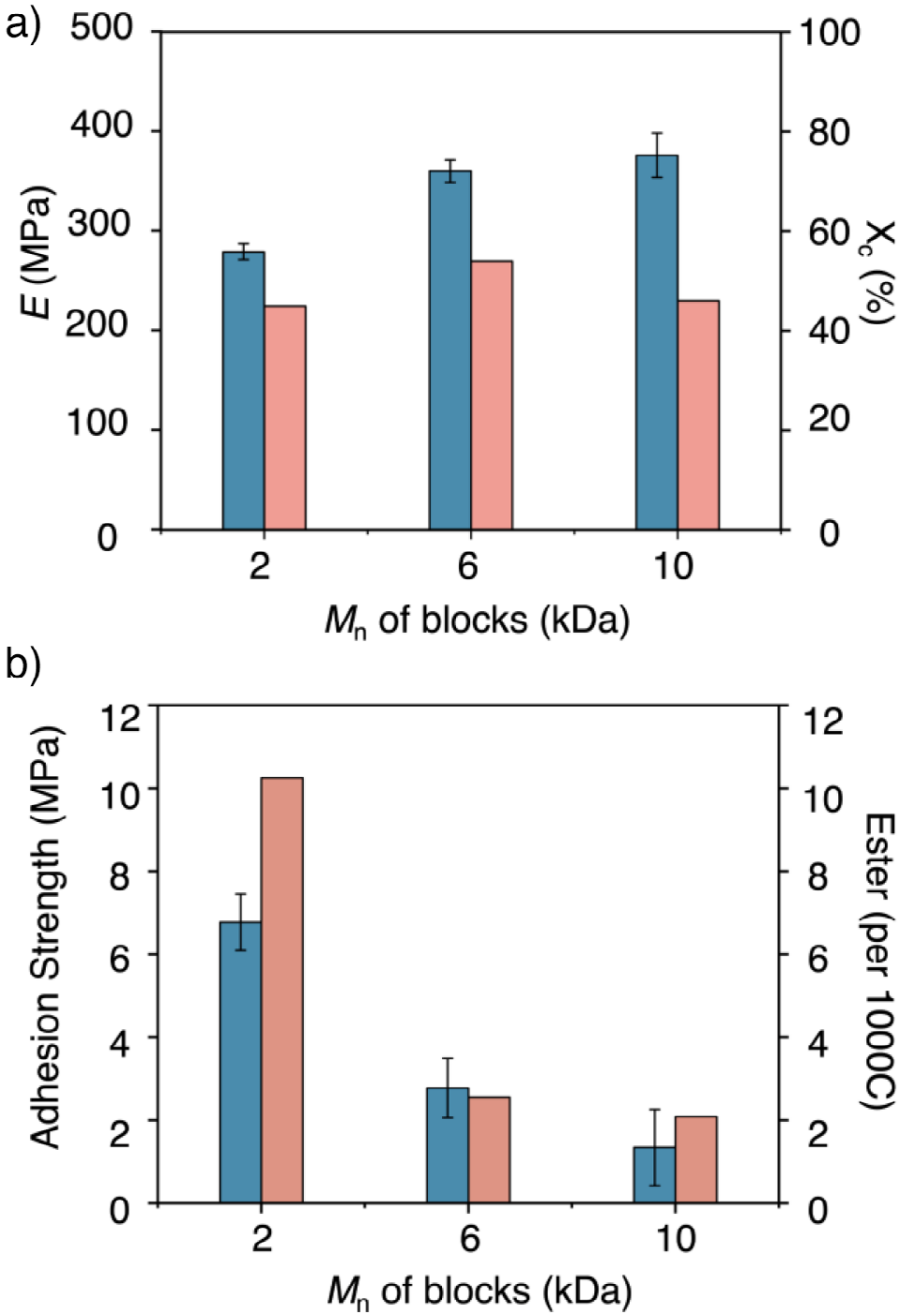
The impact of the ester content and length of building blocks on the mechanical and adhesion properties of multiblock PE‐like polymers. a) Young's modulus (blue bar) and *X*_c_ (pink bar) as a function of block molecular weight. b) Adhesion strength (blue bar) and ester content (pink bar) as a function of block molecular weight.

Multilayer films (MLFs) and multi‐material products, used especially in packaging applications,^[^ ^42^, ^43^ ^]^ present recycling challenges due to the immiscibility of their component plastics, often resulting in inefficient mechanical recycling or complex solvent recovery processes.^[^ ^44^ ^]^ Chemically recyclable adhesives offer a promising solution as tie layers that enable complete deconstruction and recovery of individual materials in MLFs. To show real‐world applications of these chemically recyclable hot‐melt adhesives, we created MLFs with HS80 as the tie layer. To fabricate MLFs, we adhered PP and Nylon together with HS80 (PP‐HS80‐Nylon) at 140 °C for 3 min (Figure 5a). Laminated glass samples were also successfully prepared. After use, the MLF was selectively depolymerized under 40 bar H_2_ at 120 °C for 24 h without additional catalyst, enabled by residual catalyst in the material, resulting in high recovery yields of PP, Nylon, and both hard and soft blocks (Figures 5b and S29–S31). Compared to strong reductants like lithium aluminum hydride, this hydrogenation‐based depolymerization is potentially more practical for real‐world applications. This high recyclability further underscores the sustainable nature of these block copolymer adhesives.

**Figure 5:**
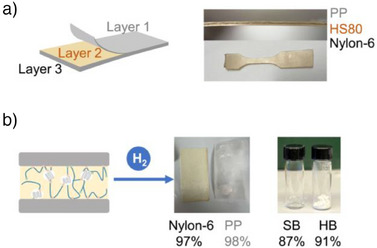
Multilayer films/materials with HS80. a) Lamination of PP and Nylon‐6. b) Lamination of glass. c) Selective depolymerization of HS80 with residual catalyst used for the multilayer films. All values are isolated yields.

In conclusion, we developed a modular set of chemically recyclable hot‐melt adhesives based on PE‐like multiblock copolymers. Soft block incorporation enabled a broad range of adhesive strengths that outperform conventional hot‐melt EVA while allowing for low and high‐temperature applications with high recyclability and reusability. Key structure–property relationships, including the roles of soft block content, ester content, and crystallinity, were uncovered, paving the way for improving recyclability and material recovery in multilayer products.

## Conflict of Interests

The authors declare no conflict of interest.

## Supporting information



Supporting Information

## Data Availability

The data that support the findings of this study are available in the Supporting Information of this article.
