# Supplementary material for: Chemically Recyclable and Tunable Polyolefin‐Like Multiblock Copolymer Adhesives
Source: Angew Chem Int Ed Engl. 2025 Sep 8;64(43):e202513286. doi: 10.1002/anie.202513286 (PMC12535384; doi:10.1002/anie.202513286)
Supplement: Supplementary file 1 — Supporting Information [file ANIE-64-e202513286-s001.pdf]

SUPPORTING INFORMATION

Chemically Recyclable and Tunable Polyolefin-like Multiblock Copolymer Adhesives

Yucheng Zhao,[a] Ethan C. Quinn,[a] Megan E. Battson,[b] Emma M. Rettner,[b] Joel Miscall,[c,d] Nicholas A. Rorrer,[c,d] Eugene Y.-X. Chen,[a] and and Garret M. Miyake*[a,b]

[a] Y. Zhao, E. C. Quinn, E. Y.-X. Chen, G. M. Miyake
Department of Chemistry
Colorado State University
Fort Collins, CO 80523, USA.
E-mail: Garret.Miyake@colostate.edu
[b] M. E. Battson, E. M. Rettner, G. M. Miyake,
School of Materials Science and Engineering
Colorado State University
Fort Collins, CO 80523, USA.
[c] J. Miscall, N. A. Rorrer
Renewable Resources and Enabling Sciences Center
National Renewable Energy Laboratory
Golden, CO 80401, USA
[d] J. Miscall, N. A. Rorrer
BOTTLE Consortium
Golden, CO 80401, USA

Table of Contents

Table of Contents 1
1. General information on material and analytical methods. 2
2. Synthesis and characterizations of multiblock polymers. 3
3. Property of adhesives 6
4. Shear strength of polymers 8
5. Multilayer materials 13
6. Supplementary spectra 16
References 17

1. General information on material and analytical methods.

Cis-cyclooctene (COE), magnesium turnings, cuprous iodide (CuI), and 1-bromohexane were obtained from Oakwood Chemicals. (H2IMes)(PPh3)(Cl)2Ru=CHPh (Grubbs II) was sourced from Umicore, and Carbonylchlorohydrido{bis[2-(diphenylphosphinomethyl)ethyl]amino}ethyl]amino}ruthenium(II) (Ru-MACHO) came from Strem Chemicals, all used without further purification. Anhydrous diethyl ether, tetrahydrofuran (THF), toluene, isopropanol, and other solvents were purchased from Sigma Aldrich or Fisher. THF and toluene were additionally purified through an mBraun MB-SPS-800 solvent purification system and stored under nitrogen. High-density polyethylene (HDPE) (Mw = 92.2 kDa), linear low-density polyethylene (LLDPE) (M_w = 90.4 kDa), and low-density polyethylene (LDPE) (M_w = 61.7 kDa), and ethylene-vinyl acetate (EVA, vinyl acetate 18 wt%, melt index 8 g/10 min (190 °C/2.16 kg)) were all acquired from Sigma Aldrich and used as received.

Nuclear Magnetic Resonance (NMR) spectra were recorded on a Bruker 400 MHz NMR Spectrometer at 298 K at 383 K. The ^{1}H NMR chemical shifts (δ, ppm) were referenced to the residual chloroform signal at 7.26 ppm in $CDCl_3$, the toluene signal at 2.09 ppm in deuterated toluene, or the tetrachloroethane signal at 6.00 ppm in deuterated tetrachloroethane.

Soft block analysis employed size exclusion chromatography (SEC) coupled with multi-angle light scattering, using an Agilent HPLC system equipped with a guard column, three PLgel 5 µm MIXED-C gel permeation columns, a Wyatt Technology TrEX differential refractometer, and a Wyatt Technology miniDAWN TREOS light scattering detector. Tetrahydrofuran (THF) served as the eluent at a flow rate of 1.0 mL/min.

High-temperature size exclusion chromatography (HT-SEC) for hard blocks and multiblock polymers was carried out on a Tosoh EcoSec HLC-8321 High Temperature SEC System with an autosampler and differential refractive index (DRI) detector. The mobile phase was 1,2,4-trichlorobenzene (TCB) (HPLC grade, Fisher Scientific). Four Tosoh TSKgel columns were employed in sequence: TSKgel guard column, TSKgel GMHHR (20) HT2, and two TSKgel G2000 columns. A TSKgel GMH HR-H (S) HT2 reference column was also used. Tosoh's Polystyrene-Quick Kit-M (PN 21916) provided polystyrene (PS) standards for calibration. Samples (6–20 mg) were placed in 10 mL high-temperature vials with PTFE caps, filtered through a 26 µm stainless steel mesh filter, and dissolved in TCB to ~1.7 mg/mL; they were then heated for two hours with occasional stirring before injection. The injection volume was 300 µL, with an operating flow rate of 1.0 mL/min for the sample columns and 0.5 mL/min for the reference column.

Differential scanning calorimetry (DSC) measurements were performed using a TA Instruments Auto Q20 in a nitrogen atmosphere, assessing T_m, T_g, and ΔH_f during the second heating cycle at 10 °C/min. Crystallinity was determined by integrating the heat of fusion and comparing it to that of fully crystalline polyethylene of similar molecular weight. Thermogravimetric analysis (TGA) was conducted with a Mettler–Toledo TGA/SDTA851, heating samples in platinum pans from room temperature to 700 °C at 10 °C/min under a nitrogen purge.

Melt-compressed films were fabricated using a Carver Auto Series Plus Laboratory Press (Model 3889.1PL1000, maximum force 15 tons) by applying 3,000 lbs of force at 150 °C for 15 minutes. The films were then cooled to ambient conditions at a rate of approximately 30 °C/min under continued compression.

Samples for lap-shear testing were prepared according to a modified version of ASTM D1002. Polymer samples were melt compressed as films between two steel plates at a force of 3000 lbs and temperature of 150 °C for 15 min. The polymer film was cut into ~ 25 × 6 mm. The film was placed between two substrates in a single lap shear configuration and then was heated to melt with a heating gun (~30 seconds). Upon cooling down to room temperature, the adhesive solidified, and the bond formed between the adhesive layer and the substrate. The specimens were clipped with a binder clip, left in a vacuum oven at 140 °C for corresponding bonding time, and then cooled down to room temperature before performing the lap shear. Adhesive strength lap shear measurements were in quintuplicate for each sample polymer using an Instron 5966 with 10kN load cell and screw side action tensile grips at a rate of 5mm/min. Substrates were 4.8mm thick aluminum sheeting, steel sheeting, polypropylene, or birch wood cut into 1”x2” strips. Adhesion strength was calculated by dividing the overlap area of the two substrates by the maximum force.

Rheological experiments were conducted on a TA Instruments Discovery HR20 rheometer equipped with a 8 mm parallel-plate geometry. Oscillatory temperature ramps were performed at a frequency of 0.1 rad/s from 30 °C to 140 °C, using a 1% strain within the linear viscoelastic regime. A 60 s hold time was applied at each temperature point before data collection. Oscillatory frequency experiments were conducted at a 1% strain from 0.01 rad/s to 100 rad/s at 25°C.

Tensile tests were conducted on an Instron 5966 universal testing system fitted with a 10 kN load cell and screw side-action tensile grips, using a crosshead speed of 5 mm/min until specimen failure. Samples were produced in accordance with ASTM D638 Type-V specifications, featuring a 3.18 mm-wide cross section.

Polarized Light Optical Microscopy was performed using a Keyence VX-6000 Digital Microscope with an insertable polarizing filter. Samples were prepared by melt pressing between glass slides at 200 °C for 30 minutes then cooled to room temperature at ~1 °C/min.

2. Synthesis and characterizations of multiblock polymers.

General procedure for synthesis of building blocks.[1]-[2]

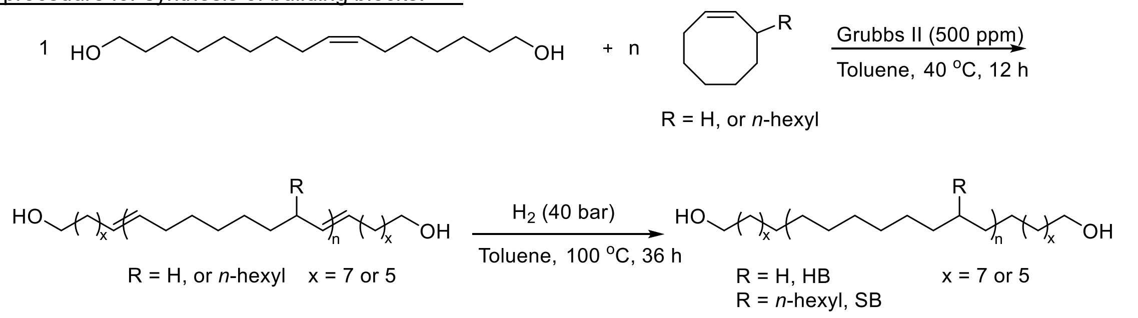

Scheme S1. The synthesis of building blocks with different molecular weight.

Inside a nitrogen-filled glove box, a stock catalyst solution was prepared by dissolving Grubbs II (1 equivalent) and *cis*-hexadec-6-ene-1,16-diol (CTA, 5 equivalents) in 1.20 mL of THF, followed by stirring for 10 minutes. In a 500 mL Schlenk flask with a stir bar, CTA (amount adjusted for the desired degree of polymerization, e.g. 20 equivalents for 10 k blocks, 33 equivalents for 6 k blocks, and 100 equivalents for 2 k blocks), monomer (2000 equivalents), and 100 mL of toluene were added. Then, 1.00 mL of the catalyst solution was introduced into the flask, which was sealed and removed from the glove box. The mixture was stirred at 40 °C for 12 hours. After cooling to room temperature, ethyl vinyl ether (100 equivalents) was added and stirred for 5 minutes to quench the reaction. A small sample was taken for ^{1}H NMR analysis, confirming over 99% conversion of CTA and monomer. The solution was then transferred to a 200 mL beaker and placed into a pressure reactor. The reactor was sealed, cycled four times with 20 bar of H_2, and then pressurized to 40 bar H_2. The mixture was heated to 100 °C and stirred at 400 rpm for 36 hours, then, the reactor was cooled to room temperature, depressurized, and flushed with nitrogen. The solution was concentrated, and the oligomers were dried under vacuum at 80 °C for 24 h. The synthesized building blocks were obtained over 90% yields and analyzed by ^{1}H NMR, SEC to give molecular weight and dispersity.

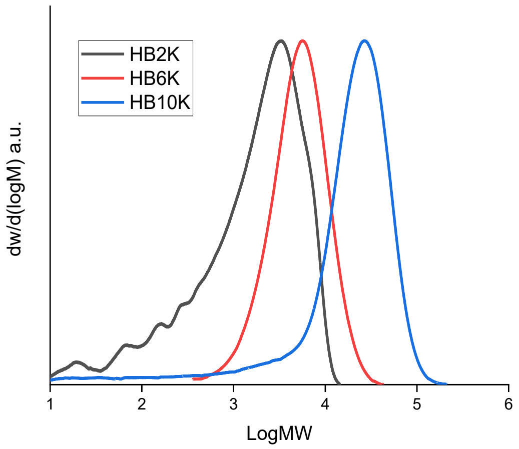

Figure S1. HT-SEC traces of HBs using TCB (160 °C). M_n (HB2k) = 1.8 kDa, Đ = 1.67; M_n (HB6k) = 7.6 kDa, *Đ* = 1.56, M_n (HB10k) = 13.7 kDa, *Đ* = 2.00.

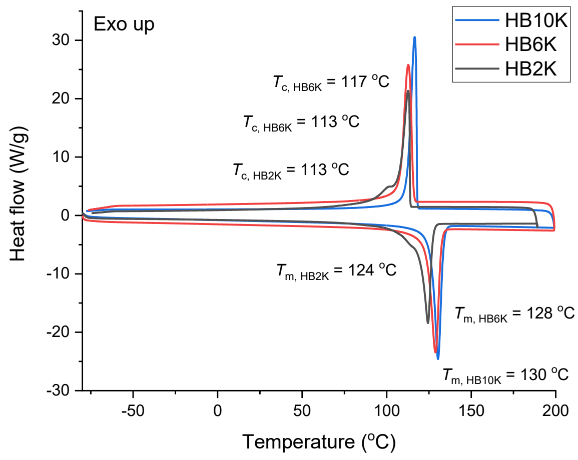

Figure S2. DSC traces of HBs with different MW. The second heat cycles were collected.

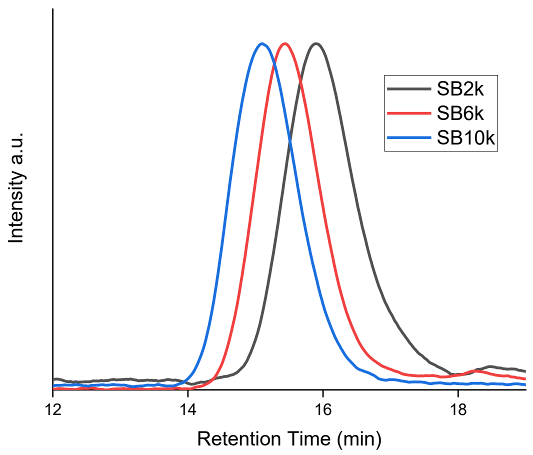

Figure S3. SEC traces of SBs (THF, 40 °C). M_n (SB2k) = 2.5 kDa, *Đ* = 1.68; M_n (SB6k) = 8.0 kDa, *Đ* = 1.29, Mn (SB10k) = 9.5 kDa, *Đ* = 1.60.

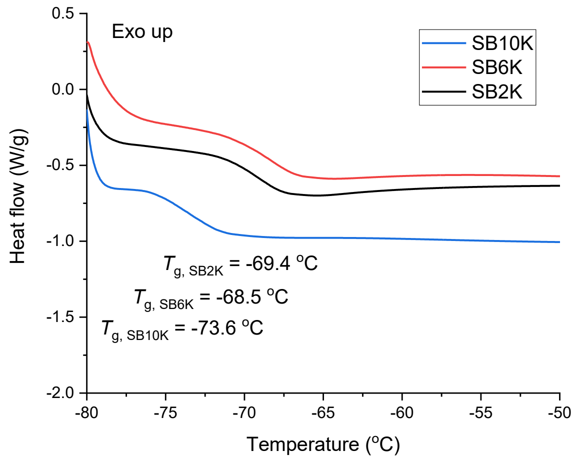

Figure S4. DSC traces of SBs with different MW. The second heat cycles were collected.

General procedure for copolymerization of multiblock copolymers.[2]

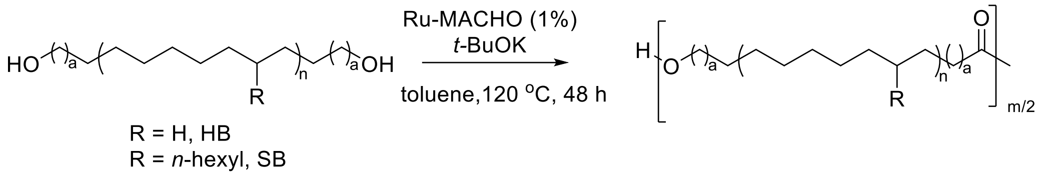

Scheme S2. The synthesis of multiblock copolymer HS0-HS100.

Preparation of catalyst solution:

In a nitrogen-filled glove box, 8.1 mg (13.0 µmol) of Ru MACHO and 1.5 mg (13.0 µmol) of potassium *tert*-butoxide were added to a 10 mL vial with a stir bar, followed by 8.1 mL of toluene. The mixture was stirred at room temperature for 10 minutes. Separately, the HB and SB components (1.00 mmol total, as detailed in Table S1) were dried in a 100 mL Schlenk tube at 130 °C under vacuum for 2 h. The tube was then transferred into the glove box, where 9.1 mg of potassium *tert*-butoxide and 6.2 mL of activated Ru-MACHO in toluene were added, followed by an additional 5.8 mL of toluene. The flask was removed and placed in an oil bath at 120 °C, and the reaction mixture was stirred for 48 h. Afterward, the mixture was diluted with 25 mL of xylenes at 140 °C, precipitated in 150 mL of isopropanol, and filtered to isolate the multiblock copolymers, which were then dried under vacuum at 80 °C for 24 h.

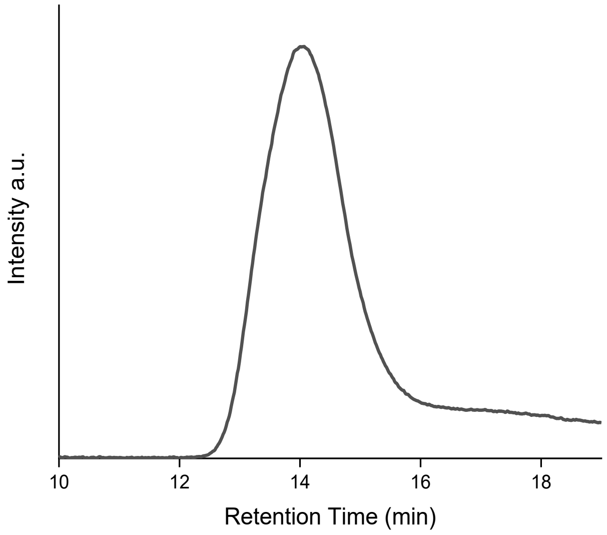

Figure S5. SEC traces of HS0 (THF as the eluent solvent).

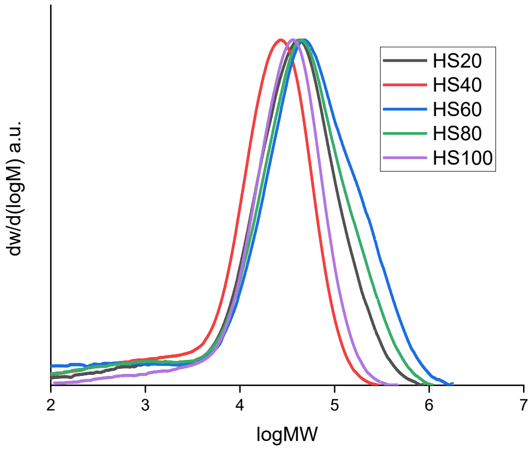

Figure S6. HT-SEC traces of HS20-HS100 using TCB (160 °C).

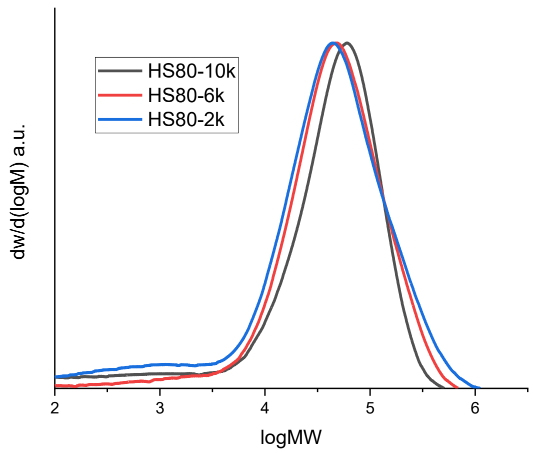

Figure S7. HT-SEC traces of HS80-10k, HS80-6k, and HS80-2k using TCB (160 °C).

3. Property of adhesives

Table S1. Mechanical and thermal properties of synthesized polymers.[a]

sample	HB (mol%)	M_w (kDa)	M_n (kDa)	$Đ$	T_m (°C)	T_g (°C)	$T_{d,5\%}$ (°C)
HS0	0	90.3	25.4	3.56	-	-59	383
HS20	20	57.5	17.2	3.33	108	-69	401
HS40	40	76.9	24.4	3.15	109	-61	407
HS60	60	87.3	22.5	3.89	113	-	408
HS80	80	82.1	21.1	3.90	118	-	414
HS100	100	57.5	18.0	3.19	123	-	417
OBC	-	81.1	41.0	2.00	-	-	-
HS80-6K	80	61.7	23.1	2.67	128	-	416
HS80-10K	80	69.2	30.0	2.31	130	-	425

[a]HS (mol%) refers to the hard segment content in the feed ratios used for copolymerization. The molecular weight parameters (M_w, M_n, and $Đ$) were determined using HT-SEC in TCB at 160 °C, with polystyrene (PS) as the standard. The T_m and T_g were analyzed using DSC. The decomposition temperature at 5% weight loss ($T_{d,5\%}$) was obtained from TGA.

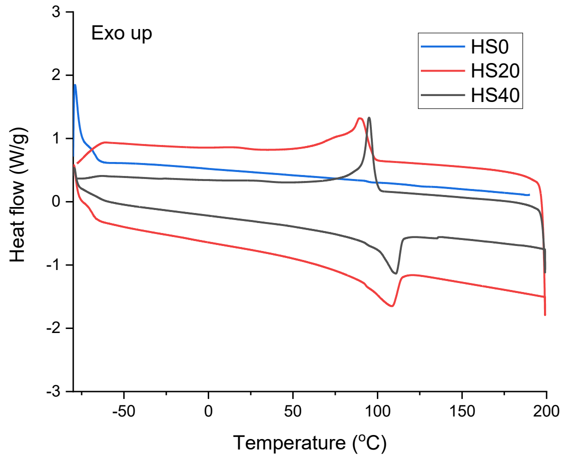

Figure S8. DSC traces of HS0, HS20, and HS40. The second heat cycles were collected.

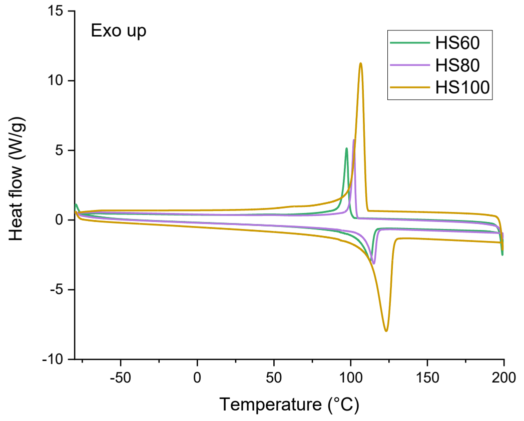

Figure S9. DSC traces of HS60, HS80, and HS100. The second heat cycles were collected.

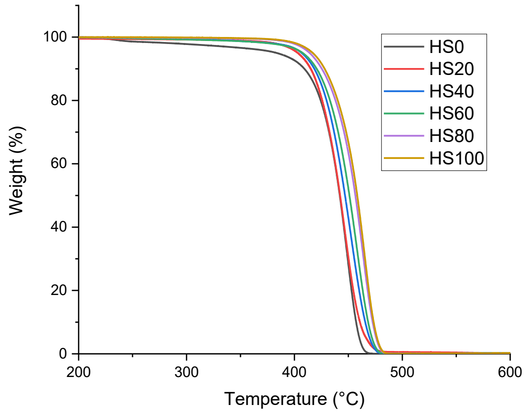

Figure S10. The TGA traces of the HS0-HS100. The $T_{d,5\%}$ for each samples were listed in Table S1.

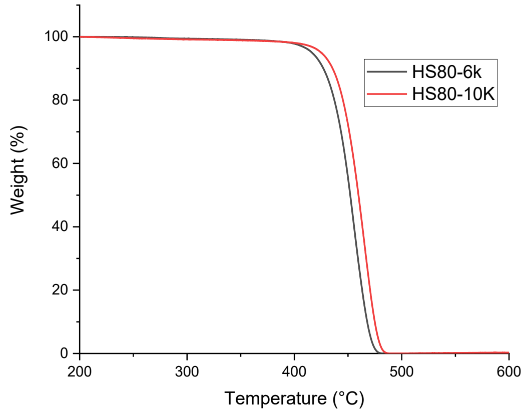

Figure S11. The TGA traces of the HS80s from different MW building blocks. HS80-6k, $T_{d,5}$ = 416 °C. HS80-10k, $T_{d,5}$ = 425 °C.

4. Shear strength of polymers

Table S2. Adhesion strength for chemically recyclable hot melt adhesive on Al surfaces.[a]

	Soft block (*mol%*)	Adhesion strength (MPa)	X_c (%)	Ester (per 1000C)
HDPE	-	0.0780±0.0140	53	-
LLDPE	-	0.0330±0.0030	32	-
LDPE	-	1.10±0.40	34	-
OBC	-	0.490±0.100	-	-
EVA		3.80±0.50	-	126
HS100	0	4.70±0.90	63	11.5
HS80	20	6.80±0.70	45	10.2
HS60	40	4.20±0.30	28	8.83
HS40	60	2.60±0.20	16	7.77
HS20	80	1.80±0.20	8.6	7.16
HS0	100	0.0420±0.080	0	7.01
HS80-6K	20	2.80±0.70	54	2.56
HS80-10K	20	1.30±0.90	46	2.08

[a]Adhesion strength for hot melt adhesives were calculated based on the area of adhesive on the aluminum surfaces and max forces of lap shear testing. X_c, degree of crystallinity of polymers were calculated based on the equation $X_c = \Delta H_f/281*100\%$ (ΔH_f for heat of fusion for fully crystalline polyethylene is 281 g^{-1} of relevant molecular weight (M_w = 60.7 kDa). The ester content (%) was calculated based on the ratio of ester groups to total carbon content, determined from the ^{1}H NMR spectra (Figures S32-S38) and corroborated with literature values.[2]

Table S3. Adhesion strength for HS80 on different surfaces.

	Al	steel	PP	HDPE[a]	PVC	wood
Adhesion strength (MPa)	6.80±0.70	6.40±1.70	1.20±0.10	1.00±0.50	0.13±0.02	7.40±0.60

[a]Samples were prepared at bonding temperature of 120 °C.

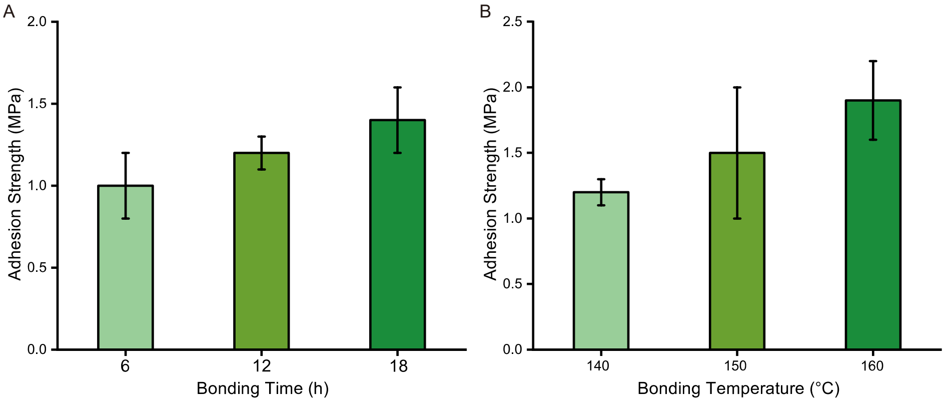

Figure S12. Adhesion strength of HS80 on PP surface A) at different bonding times from 6 – 18 h, and B) at bonding temperature of 140 – 160 °C.

Table S4. Adhesion strength of EVA and HS80 on aluminum after treatment at various temperatures.[a]

	22° C	-196° C
Adhesion strength for EVA	4.70±0.90	0.47±0.20
Adhesion strength for HS80	6.80±0.70	4.90±1.60

[a]Samples were immersed in liquid nitrogen for 1 h and measured immediately afterward.

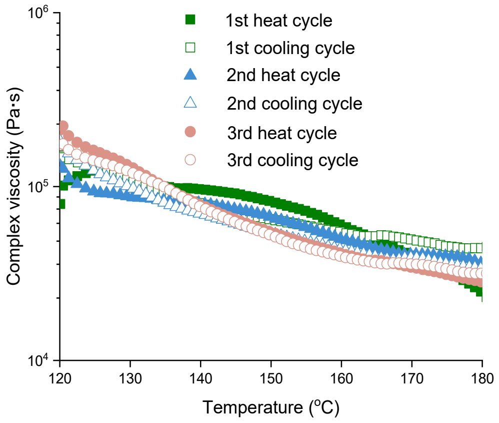

Figure S13. Complex viscosity of HS80 under three heat-cooling cycles.

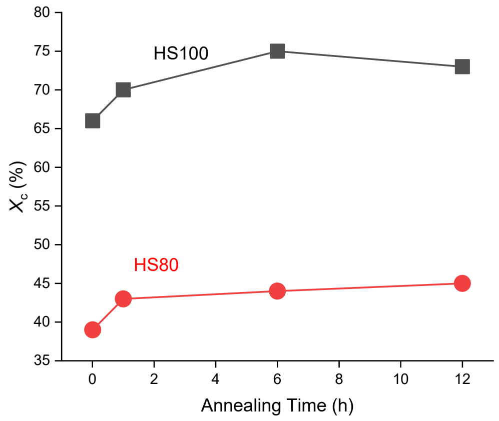

Figure S14. X_c as a function of annealing time at 110 °C.

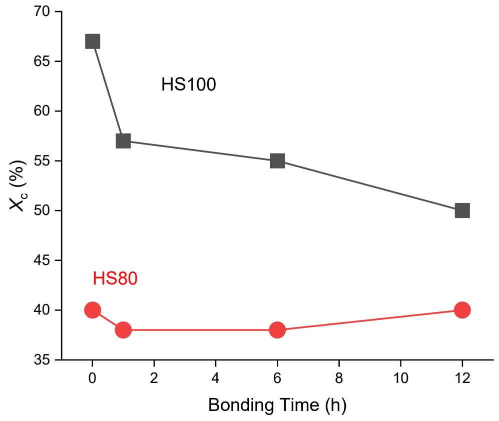

Figure S15. X_c as a function of bonding time at 140 °C. The X_c of HS80 remained nearly constant across all time points, suggesting a rapid crystallization rate and efficient crystal reformation. In contrast, the X_c of HS100 gradually decreased from 67% to 50% as bonding time increased from 5 min to 12 h. This reduction is likely due to the melting and disruption of smaller or less stable crystallites during prolonged thermal exposure.

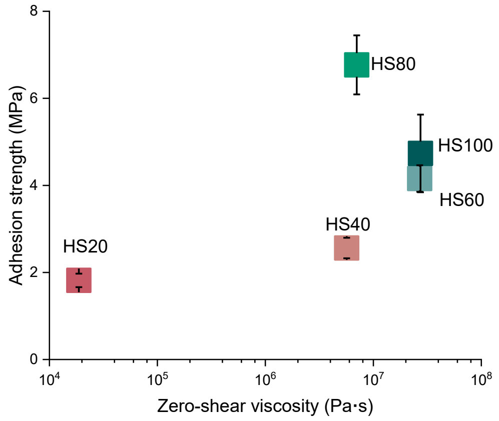

Figure S16. Adhesion strength as a function of zero-shear viscosity for HS20-HS100.

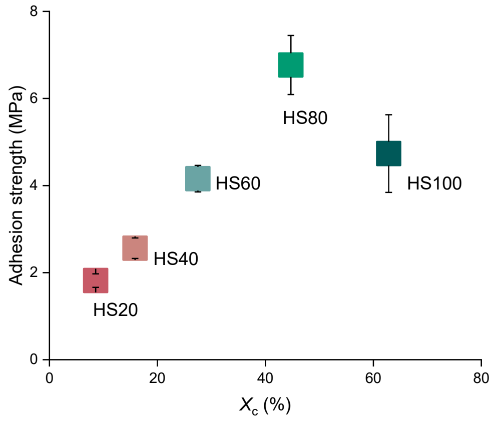

Figure S17. Adhesion strength as a function of degree of crystallinity (X_c) for HS20-HS100.

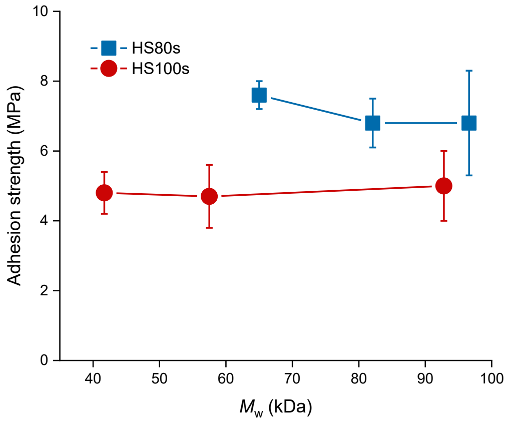

Figure S18. Adhesion strength on aluminum as a function of the weight-average molecular weight (M_w) for HS80 and HS100. For HS80, the adhesion strengths were 7.60 MPa, 6.80 MPa, and 6.80 MPa at M_w values of 65.0 kDa, 82.1 kDa, and 96.6 kDa, respectively. For HS100, the adhesion strengths were 4.80 MPa, 4.70 MPa, and 5.00 MPa at M_w values of 41.7 kDa, 57.5 kDa, and 92.8 kDa, respectively.

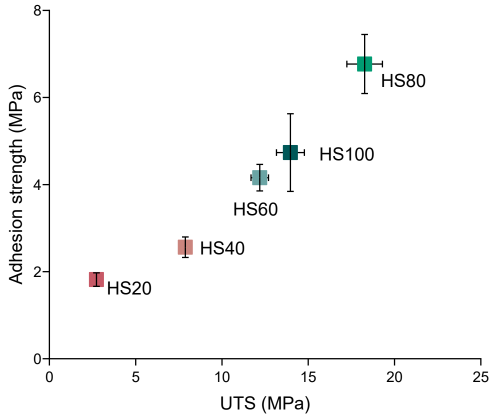

Figure S19. Adhesion strength as a function of ultimate tensile strength for HS20-HS100.

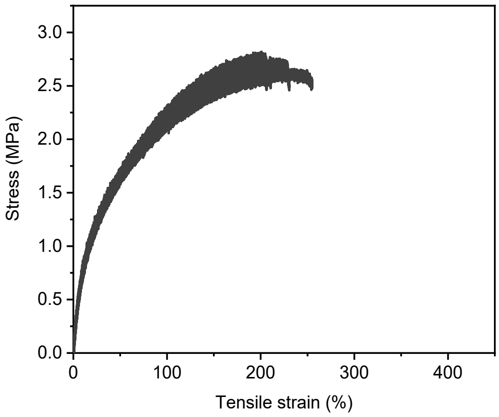

Figure S20. Stress-strain curves of HS20 (5mm/min).

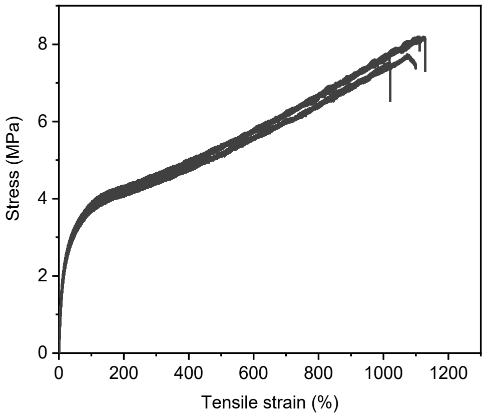

Figure S21. Stress-strain curves of HS40 (5mm/min).

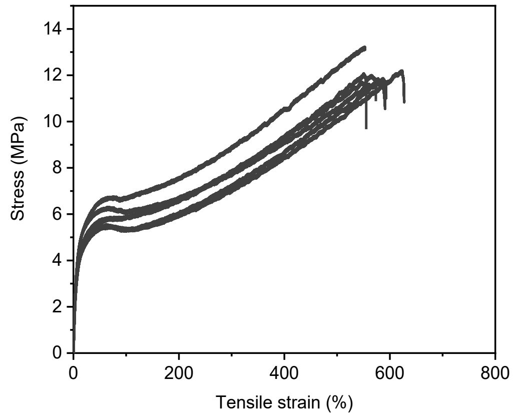

Figure S22. Stress-strain curves of HS60 (5mm/min).

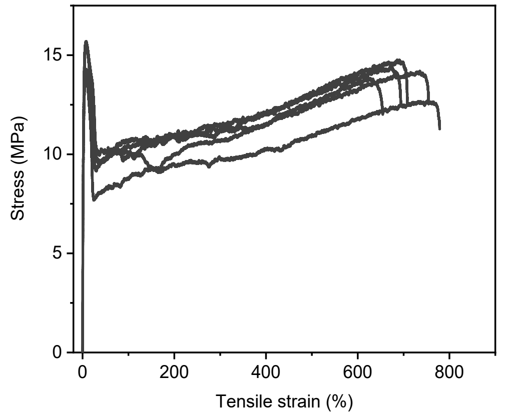

Figure S23. Stress-strain curves of HS100 (5mm/min).

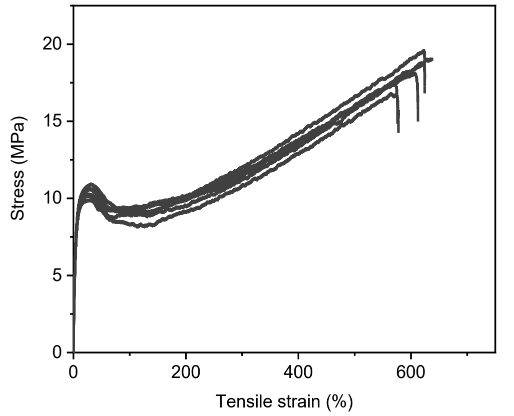

Figure S24. Stress-strain curves of HS80 (5mm/min).

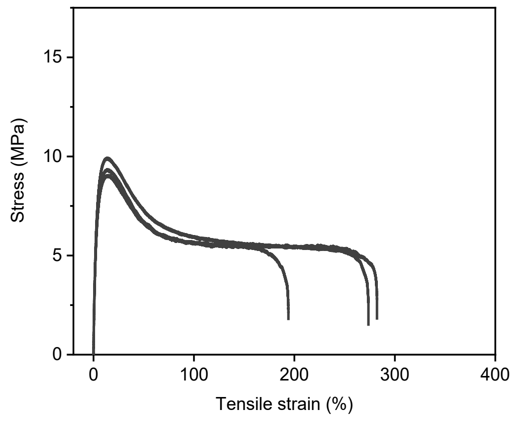

Figure S25. Stress-strain curves of HS80-6k (5mm/min).

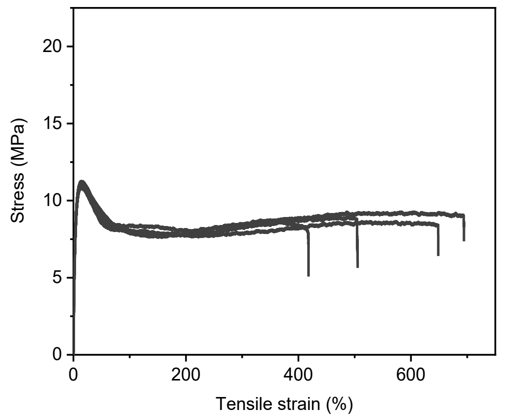

Figure S26. Stress-strain curves of HS80-10k (5mm/min).

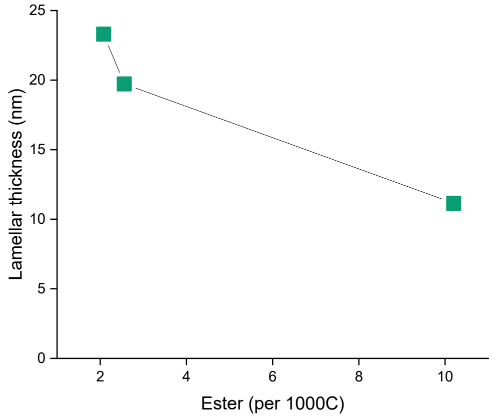

Figure S27. The calculated lamellar thicknesses as a function of the mole fraction of ester groups of multiblock PE80s. Lamellar thicknesses were estimated using the T_m values of HS80s from Table S1 and the Gibbs–Thomson equation.

Gibbs−Thomson equation:

$$T_m = T_m^0\left(1-\frac{2\sigma}{L_m \Delta H_f^0}\right)$$

Where T_m^0 is the melting temperature of an extended crystal, σ is the crystalline/amorphous interfacial energy, ΔH_f^0 is the heat of fusion per volume of crystal. T_m^0 = 414 K, σ = 8.7 μJ·cm^{-2}, ΔH_f^0 = 281 J g^{-1}.[3]

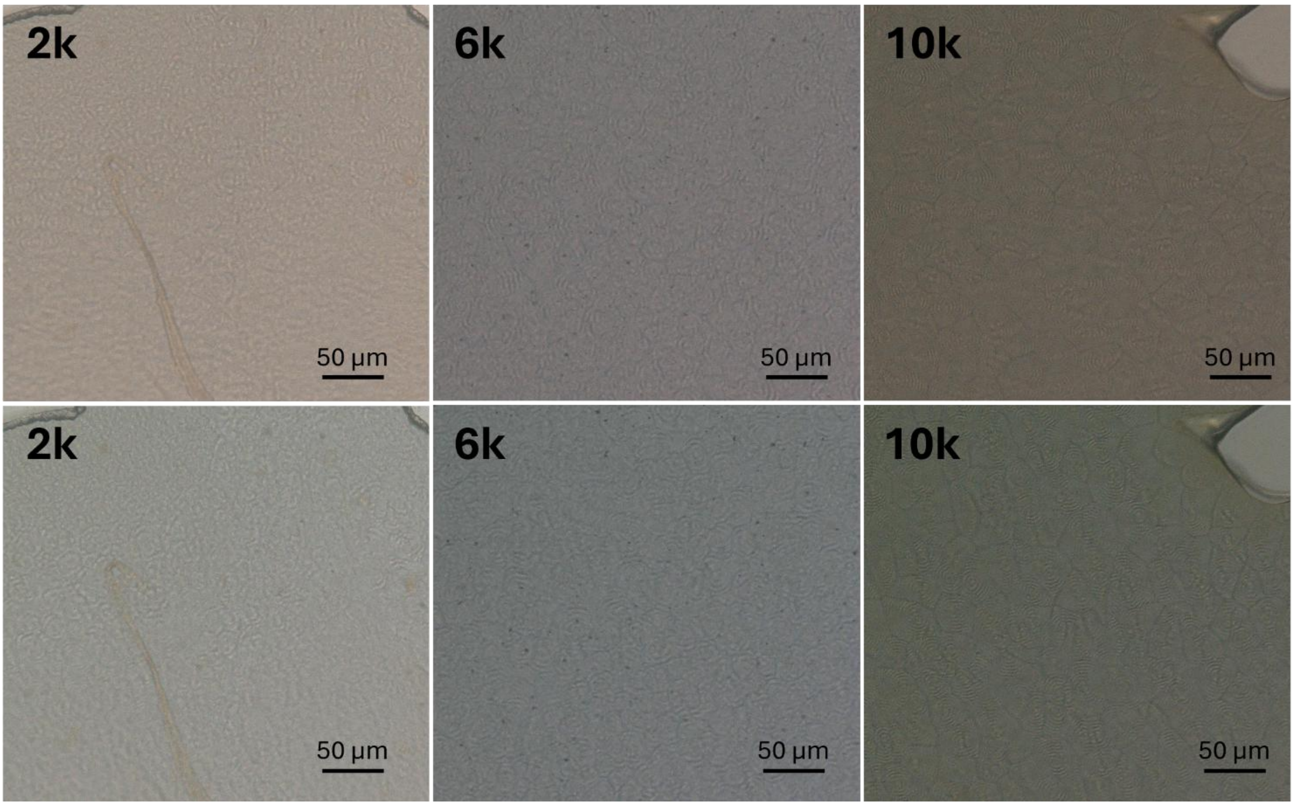

Figure S28. Polarized Light Optical Micrographs of HS80 samples showing increasing crystal size with increasing distance between esters. From left to right, birefringence becomes more obvious and increases in diameter from sub-10 μm to ~50 μm. Top and bottom images show identical locations under perpendicular directions of polarized light.

5. Multilayer materials

General procedure for lamination

Prior to lamination, a PP film (0.40 mm thick), a Nylon film (0.40 mm thick), and an HS80 film (0.20 mm thick, 108 mg) were prepared. The films were then assembled into a multilayer structure and processed in a hot press at 3000 lbs for 3 minutes. Finally, the laminated assembly was cooled to room temperature using water cooling.

General procedure for selective depolymerization

The residual catalyst in the copolymers was shown to catalyze the hydrogenation of ester groups in the main chain to alcohols under high temperature and H_2, enabling selective depolymerization of hot-melt adhesives in laminated materials without the need for additional catalyst—an advantage for real-world applications.

In a nitrogen-filled glovebox, a laminated film (containing 108 mg HS80), 4.0 mg (0.036 mmol) of potassium tert-butoxide, and 25.0 mL of toluene were added to a 100 mL beaker with a stir bar. The beaker was then removed from the glovebox and placed in a pressure reactor. After sealing, the reactor was cycled four times with 20 bar H_2 and then charged with 40 bar H_2 before being heated at 120 °C for 24 h. Following cooling to room temperature, the reactor was depressurized and flushed with nitrogen. The undissolved PP and Nylon films were removed and washed with toluene, and the remaining solution was filtered; the solid was subsequently washed with hexane to yield 71.8 mg HB. Upon concentration, 23.5 mg SB was obtained with an 87% yield.

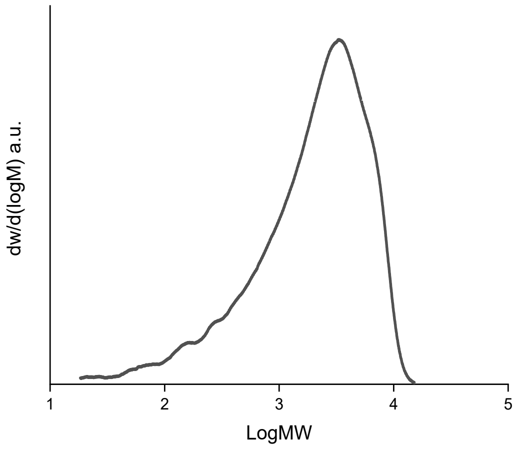

Figure S29. HT-SEC trace of HB2k after depolymerized using TCB (160 °C). M_n = 1.9 kDa, $Đ$ = 1.73.

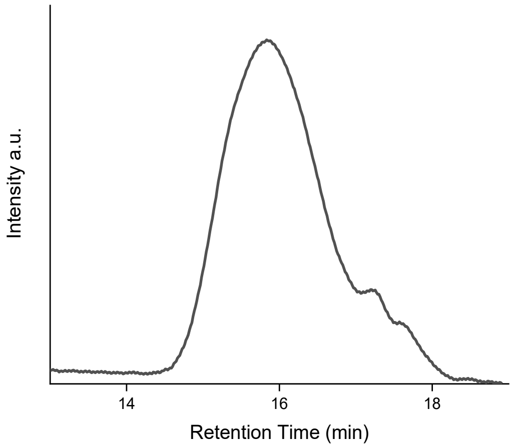

Figure S30. SEC trace of SB2k after depolymerized using THF (40 °C). M_n = 2.3 kDa, $Đ$ = 1.71.

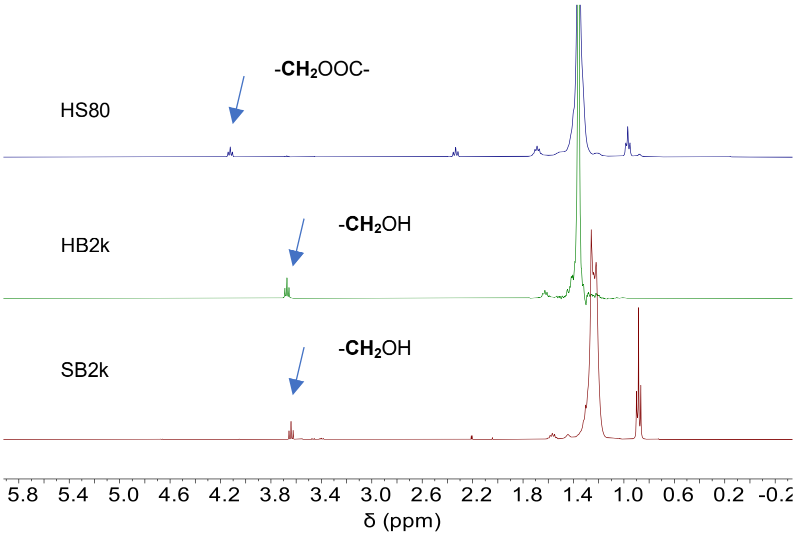

Figure S31. ^{1}H NMR spectra of HS80 (Blue; 400 MHz TCE-d$_2$, 383 K), HB2k after depolymerization (Green; 400 MHz TCE-d$_2$, 383 K), and SB2k after depolymerization (Red; 400 MHz, $CDCl_3$, 298 K).

6. Supplementary spectra

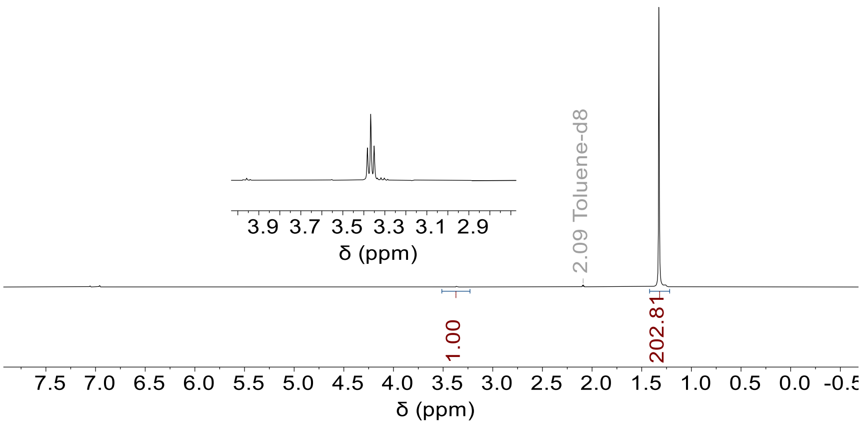

Figure S32. ^{1}H NMR spectra of HB-6k (400 MHz, toluene-d8, 373 K).

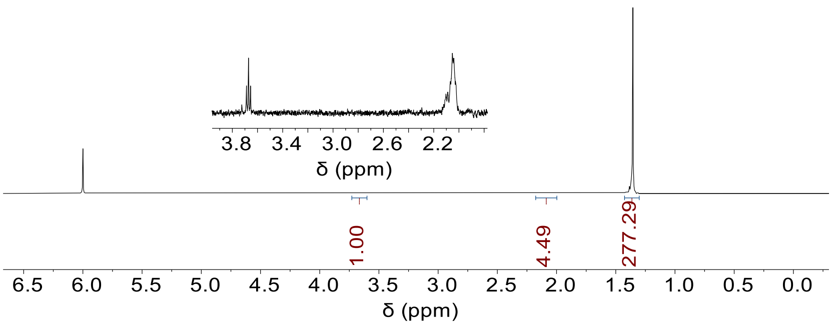

Figure S33. ^{1}H NMR spectra of HB-10k (400 MHz, TCE-d2, 383K).

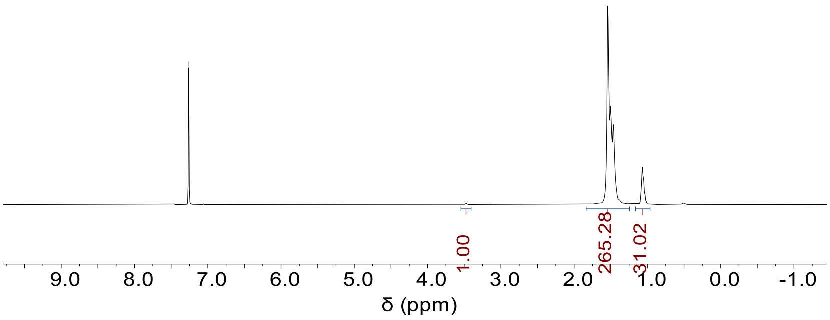

Figure S34. ^{1}H NMR spectrum of SB-6k (400 MHz, $CDCl_3$, 298 K).

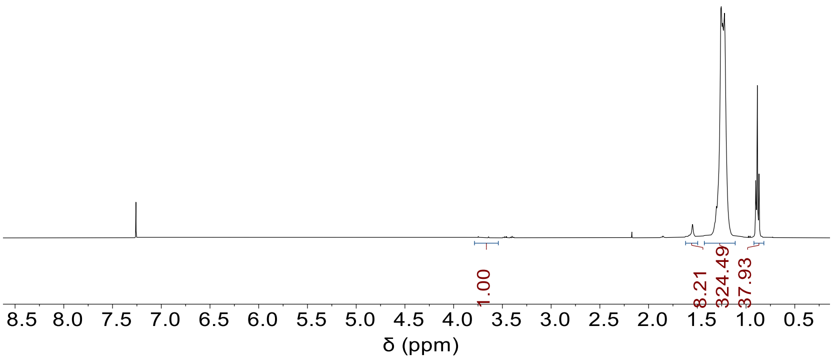

Figure S35. ^{1}H NMR spectrum of SB-10k (400 MHz, $CDCl_3$, 298 K).

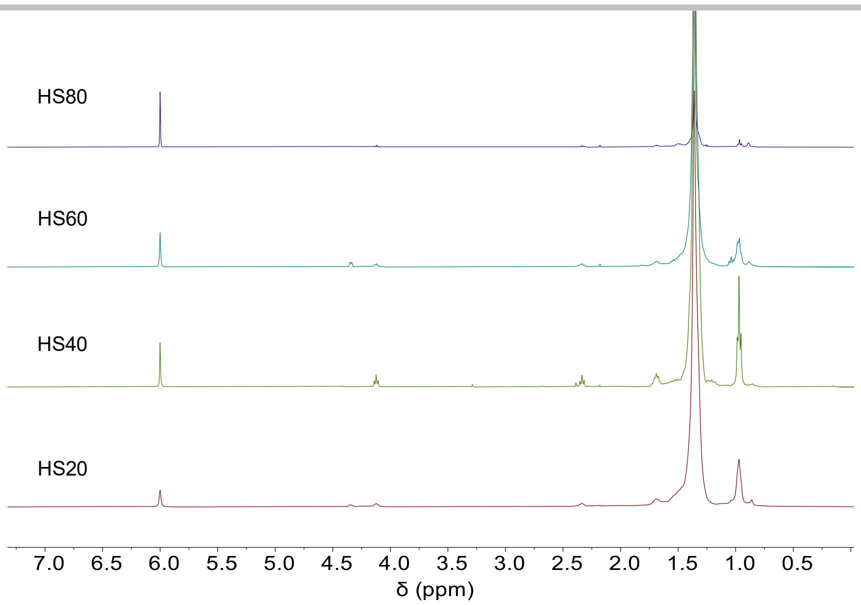

Figure S36. ^{1}H NMR spectra of HS20-HS80 (400 MHz, TCE-d2, 383 K).

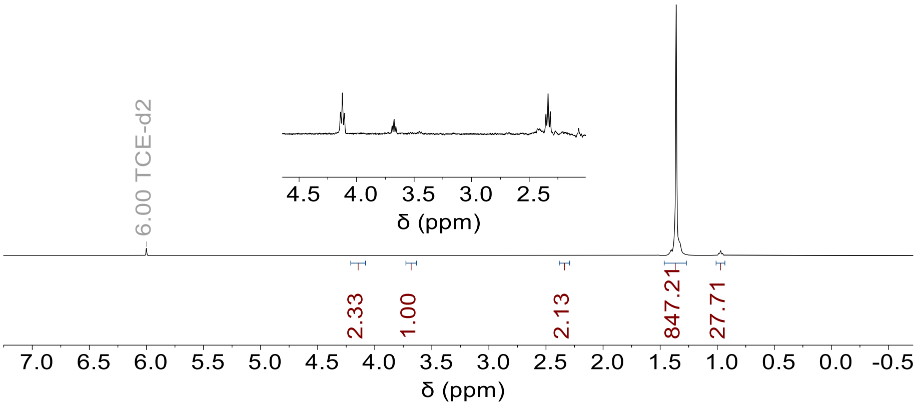

Figure S37. ^{1}H NMR spectra of HS80-6k (400 MHz, TCE-d2, 383 K).

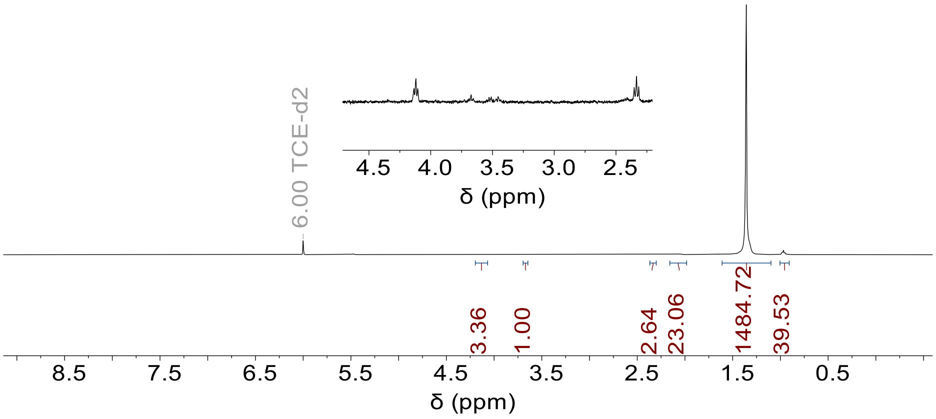

Figure S38. ^{1}H NMR spectra of HS80-10k (400 MHz, TCE-d2, 383 K).

References

[1] a) S. Kobayashi, L. M. Pitet, M. A. Hillmyer, *J. Am. Chem. Soc.* **2011**, *133*, 5794-5797; b) C. S. Sample, E. A. Kellstedt, M. A. Hillmyer, *ACS Macro. Lett.* **2022**, *11*, 608-614.
[2] Y. Zhao, E. M. Rettner, K. L. Harry, Z. Hu, J. Miscall, N. A. Rorrer, G. M. Miyake, *Science* **2023**, *382*, 310-314.
[3] M. P. F. Pepels, M. R. Hansen, H. Goossens, R. Duchateau, *Macromolecules* **2013**, *46*, 7668-7677.
